# Intrinsic subtypes in Ethiopian breast cancer patient

**DOI:** 10.1007/s10549-022-06769-z

**Published:** 2022-10-25

**Authors:** Zelalem Desalegn, Meron Yohannes, Martin Porsch, Kathrin Stückrath, Endale Anberber, Pablo Santos, Marcus Bauer, Adamu Addissie, Yonas Bekuretsion, Mathewos Assefa, Yasin Worku, Lesley Taylor, Tamrat Abebe, Eva Johanna Kantelhardt, Martina Vetter

**Affiliations:** 1 Department of Microbiology, Immunology, and Parasitology, School of Medicine, Addis Ababa University, Addis Ababa, Ethiopia; 2 Global Health Working Group, Martin Luther University Halle-Wittenberg, Halle (Saale), Germany; 3 School of Medical Laboratory Sciences, Addis Ababa University, Addis Ababa, Ethiopia; 4 Institute of Computer Science, Martin Luther University Halle-Wittenberg, Halle (Saale), Germany; 5 Department of Gynecology, Martin Luther University Halle-Wittenberg, Halle (Saale), Germany; 6 Department of Surgery, School of Medicine, Addis Ababa University, Addis Ababa, Ethiopia; 7 Institute of Medical Epidemiology, Biostatistics and Informatics, Martin Luther University Halle-Wittenberg, Halle (Saale), Germany; 8 Institute of Pathology, Martin Luther University Halle-Wittenberg, Halle (Saale), Germany; 9 School of Public Health, College of Health Sciences, Addis Ababa University, Addis Ababa, Ethiopia; 10 Department of Pathology, School of Medicine, Addis Ababa University, Addis Ababa, Ethiopia; 11 Department of Oncology, School of Medicine, Addis Ababa University, Addis Ababa, Ethiopia; 12 College of Medicine and Health Science, Wollo University, Dessie, Wollo Ethiopia; 13 City of Hope National Medical Center, Duarte, CA USA

**Keywords:** Ethiopia, Breast cancer, PAM50, Intrinsic subtyping

## Abstract

**Purpose:**

The recent development of multi-gene assays for gene expression profiling has contributed significantly to the understanding of the clinically and biologically heterogeneous breast cancer (BC) disease. PAM50 is one of these assays used to stratify BC patients and individualize treatment. The present study was conducted to characterize PAM50-based intrinsic subtypes among Ethiopian BC patients.

**Patients and methods:**

Formalin-fixed paraffin-embedded tissues were collected from 334 BC patients who attended five different Ethiopian health facilities. All samples were assessed using the PAM50 algorithm for intrinsic subtyping.

**Results:**

The tumor samples were classified into PAM50 intrinsic subtypes as follows: 104 samples (31.1%) were luminal A, 91 samples (27.2%) were luminal B, 62 samples (18.6%) were HER2-enriched and 77 samples (23.1%) were basal-like. The intrinsic subtypes were found to be associated with clinical and histopathological parameters such as steroid hormone receptor status, HER2 status, Ki-67 proliferation index and tumor differentiation, but not with age, tumor size or histological type. An immunohistochemistry-based classification of tumors (IHC groups) was found to correlate with intrinsic subtypes.

**Conclusion:**

The distribution of the intrinsic subtypes confirms previous immunohistochemistry-based studies from Ethiopia showing potentially endocrine-sensitive tumors in more than half of the patients. Health workers in primary or secondary level health care facilities can be trained to offer endocrine therapy to improve breast cancer care. Additionally, the findings indicate that PAM50-based classification offers a robust method for the molecular classification of tumors in the Ethiopian context.

**Supplementary Information:**

The online version contains supplementary material available at 10.1007/s10549-022-06769-z.

## Introduction

Breast cancer (BC) is one of the most commonly diagnosed cancer worldwide with an estimated 2.3 million new cases in 2020. Improved screening, systemic drug treatment such as endocrine therapy, chemotherapy, targeted therapy, immunotherapy, and surgery, radiotherapy have contributed significantly in reducing mortality [1]. In the resource-limited settings, BC incidence and mortality tend to increase [2]. In the context of Sub-Saharan Africa, access to diagnosis and cancer care have been identified as important determinants for survival [3]. In Ethiopia, BC is one of the most common cancers [4]. Previous survival studies demonstrated that women in Ethiopia had a favorable 5-year outcome with 45% metastasis free survival [5]. In another study in rural Ethiopia, the 2-year overall survival was only 53% [6].

Prognostic and predictive factors such as age, tumor size, nodal status, grading, Ki-67 proliferation index, angio-invasion, hormone receptor (HR) status, human epidermal growth factor receptor 2 (HER2) status are used in the clinical routine for prognosis of the patient and predicting benefit from therapy [7]. In addition, molecular biomarkers have an increasingly important role tailoring the individualized treatment recommendation for BC patients in the selection of chemotherapy, endocrine or immunotherapy regimen [8].

Gene expression analysis has helped identify distinct molecular signatures in breast cancer that have different prognostic outcomes in addition to clinical and histopathological features. Multi-gene assays in early breast cancer are now routinely used in clinical practice and are integrated into national and international guidelines [9]. Parker and colleagues simplified the profiling algorithms using 50 genes for classification in one of the intrinsic subtypes: luminal A, luminal B, HER2-enriched, and basal-like [10, 11]. The PAM50 classifier provides in addition to traditional clinical and histopathological biomarkers prognostic and predictive value for BC patients [11].

Studies from different regions in Africa have reported proportions of estrogen receptor-positive disease varying between 20 and 70% supposedly due to differences in genetic background, tumor size, but also quality issues have been discussed [12]. Our own data from Ethiopia showed 65% estrogen receptor-positive tumors assessed by immunohistochemistry [13]. The present study was conducted to assess the performance of the PAM50 classifier in Ethiopian breast cancer specimens and to assess associations of the intrinsic subtypes with protein expression of estrogen receptor, progesterone receptor and HER2.

## Patients and methods

### Patients and samples

All female patients with invasive carcinoma of the breast were included in this study in accordance with the REMARK criteria [14]. Formalin-fixed paraffin-embedded (FFPE) tissues from pathologically confirmed BC samples (*n* = 334) were collected by ZD and MY from different hospitals and pathology laboratories in Ethiopia (Tikur Anbessa Specialized Hospital, Zewditu Memorial Hospital, Yekatit 12 Hospital, Bethzatha General Hospital, St. Paul’s Hospital Millennium Medical College, Aira General Hospital). Clinical and histopathological data including age at diagnosis, tumor size, histologic type and tumor differentiation were retrieved from patient’s medical records. An overview of all parameters considered here is given in the Supplementary Table S1.

### IHC and gene expression analysis

FFPE tissue was employed for the analysis of the expression of ER, PgR, HER2, and Ki-67 using immunohistochemistry (IHC). IHC analyses were carried out using specific antibodies according to the manufacturers’ instructions as follows:

- ER: Clone Ab-11; Thermo Scientific, MA; USA, Catalog Number MS-354-P1, mouse host, dilution 1:150
- PgR: Clone PgR 636; DAKO, CA, USA, Catalog Number M3569, mouse host, dilution 1:100
- HER2: Clone DG44; DAKO; CA, USA, Catalog Number SK001, rabbit host, ready to use
- Ki-67: Clone SP6; Thermo Scientific; MA, USA, Catalog Number RM-9106-S; mouse host; dilution 1:250

Expression of HR and HER2 status was performed according to the current guidelines [15], positivity of ER and PgR status was declared when the IRS was > 0. If at least one of the markers were positive, the HR status was defined as positive. HER2 status was assessed according to ASC-CAP guidelines [16], HER2 DAKO 3 was considered positive, HER2 DAKO 0 and DAKO 1 was considered HER2-negative. Tissue with HER2 DAKO 2 was interpreted as equivocal and cromogen in situ hybridization (CISH) was performed for confirmation. Based on previous reports, Ki-67 proliferation index was graded as ‘low’ if Ki-67 staining was positive in < 20% of tumor cells or ‘high’ when at least 20% of tumor cells stained positive [17, 18]. The histological grading was performed using Elston–Ellis grading system [19].

After identification of the tumor areas on HE-stained slides by the pathologist, 3–5 adjacent unstained tumor slides (10 µm) were processed using miRNeasy FFPE Mini Kit® (Qiagen) according to the manufacturer’s protocol. The extracted RNA concentration and quality was measured using Nanophotometer.

Relative gene expression was measured using the NanoString nCounter® Analysis System (NanoString Technologies, Seattle, WA, USA) using a multiplexed hybridization assay, digital readouts of fluorescent barcoded probes which hybridize with each mRNA sequence of interest. The data collection was carried out in the nCounter® Digital Analyzer. Data import, quality control, and normalization of expression levels were conducted with the nSolver software version 4 (NanoString Technologies, Inc.). Background subtraction from raw transcript counts was performed through negative input controls. Following reference-normalization by dividing the geometric mean of six references-control genes (*ACTB*, *G6PD*, *RPLP0*, *TBP*, *TFRC* and *UBB*), normalized counts were log2-transformed prior to all downstream analyses. Intrinsic subtype classification was calculated using the nearest PAM50 centroid algorithm Bioclassifier and NanoStringNorm implemented in R [11, 20].

### Endpoints and statistical analysis

As a first objective, the distribution of the intrinsic subtypes within an Ethiopian cohort was defined. The secondary objectives were the associations of the intrinsic subtypes to clinical and histopathological parameters including the IHC groups, applying logistic regression. In order to tackle multiple testing, we reduced the subgroup analyses to pre-defined, well-accepted and clinical relevant groups (e.g., age, tumor size, tumor grade/differentiation, ER status, PgR status, HER2 status, Ki-67 proliferation index and IHC groups). Statistical significance was declared for *p*-values < 1% (two-sided Pearson’s chi-square tests for independence, with Yates’ correction for continuity when relevant). Statistical analyses were carried out using SPSS 25 (IBM, Armonk NY, USA).

## Results

### Distribution of intrinsic subtypes

**Fig. 1:**
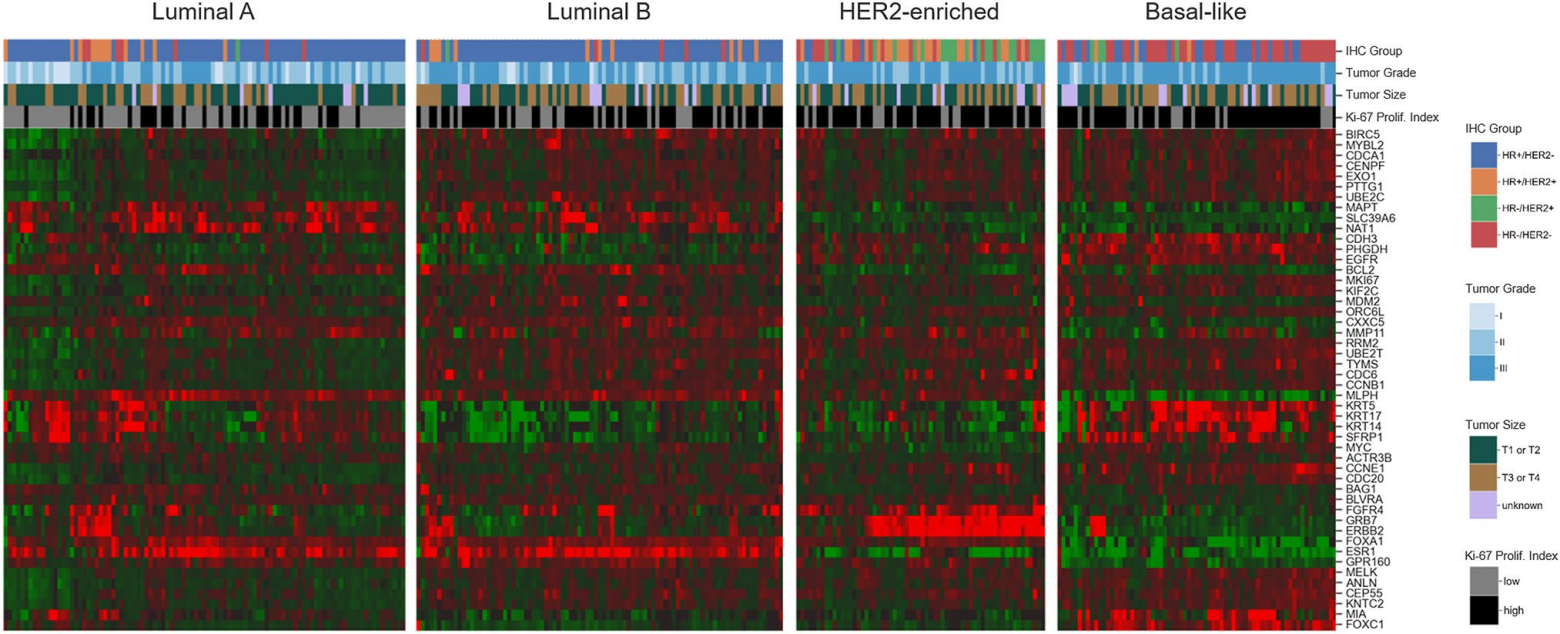
Gene Expression heatmap of the 50 loci used for the PAM50 classification of 334 BC samples. The 334 samples are grouped horizontally according to their intrinsic subtype, which are indicated above each block. Red tiles denote overexpression, while green tiles correspond to underexpression. The four horizontal bars above the heatmaps indicate the classification of samples according to IHC groups, tumor grade, tumor size and Ki-67 proliferation index (top-down, respectively, with color codes for each bar given at the right side of the figure). *HR* hormone receptor, *HER2* human epidermal growth factor receptor 2

The classification of intrinsic subtypes based on the PAM50 assay yielded 104 luminal A (31.1%), 91 luminal B (27.2%), 62 HER2-enriched (18.6%) and 77 basal-like samples (23.1%). The expression levels of the 50 loci included in the assay (Fig. 1) revealed a gene expression signature which was unique for each subtype. An overview of the distribution of selected clinical and histopathological parameters among the PAM50-based intrinsic subtypes is given in Table 1.

**Table 1:** Distribution of clinical and histopathological parameters among intrinsic subtypes

Parameters	All *n* = 334	Luminal A *n* = 104	Luminal B *n* = 91	HER2−enriched *n* = 62	Basal-like *n* = 77	*p*-value
Age group (years)						0.67
< 50	201 (60.2%)	66 (63.5%)	58 (63.7%)	33 (53.2%)	44 (57.1%)	
≥ 50	95 (28.4%)	29 (27.9%)	24 (26.4%)	21 (33.9%)	21 (27.3%)	
Unknown	38 (11.4%)	9 (8.7%)	9 (9.9%)	8 (12.9%)	12 (15.6%)	
Tumor size						0.01
T1 or T2	168 (50.3%)	68 (65.4%)	39 (42.9%)	31 (50.0%)	30 (39.0%)	
T3 or T4	126 (37.7%)	29 (27.9%)	41 (45.1%)	23 (37.1%)	33 (42.9%)	
Unknown	40 (12.0%)	7 (6.7%)	11 (12.1%)	8 (12.9%)	14 (18.2%)	
Histological types						0.45
NST	303 (90.7%)	94 (90.4%)	86 (94.5%)	54 (87.1%)	69 (89.6%)	
Non-NST	31 (9.3%)	10 (9.6%)	5 (5.5%)	8 (12.9%)	8 (10.4%)	
Tumor grade*						**3.35 × 10**^**–11**^
G1 or G2	140 (41.9%)	71 (68.3%)	37 (40.7%)	18 (29.0%)	14 (18.2%)	
G3	194 (58.1%)	33 (31.7%)	54 (59.3%)	44 (71.0%)	63 (81.8%)	
Estrogen receptor status*						**5.11 × 10**^**–16**^
Positive (≥ 1%)	184 (55.1%)	77 (74.0%)	69 (75.8%)	18 (29.0%)	20 (26.0%)	
Negative (< 1%)	150 (44.9%)	27 (26.0%)	22 (24.2%)	44 (71.0%)	57 (74.0%)	
Progesterone receptor status*						**6.99 × 10**^**–15**^
Positive (≥ 1%)	157 (47.0%)	69 (66.3%)	60 (65.9%)	13 (21.0%)	15 (19.5%)	
Negative (< 1%)	177 (53.0%)	35 (33.7%)	31 (34.1%)	49 (79.0%)	62 (80.5%)	
Hormone receptor status*						**4.43 × 10**^**–25**^
Positive	232 (69.5%)	95 (91.3%)	85 (93.4%)	26 (41.9%)	26 (33.8%)	
Negative	102 (30.5%)	9 (8.7%)	6 (6.6%)	36 (58.1%)	51 (66.2%)	
HER2 status*						**7.56 × 10**^**–19**^
Negative	261 (78.1%)	91 (87.5%)	80 (87.9%)	21 (33.9%)	69 (89.6%)	
Positive	73 (21.9%)	13 (12.5%)	11 (12.1%)	41 (66.1%)	8 (10.4%)	
Ki-67 proliferation index*						**2.48 × 10**^**–8**^
Low (< 20%)	132 (39.5%)	66 (63.5%)	31 (34.1%)	17 (27.4%)	18 (23.4%)	
High (≥ 20%)	202 (60.5%)	38 (36.5%)	60 (65.9%)	45 (72.6%)	59 (76.6%)	

*HER2* human epidermal growth factor receptor 2, *NST* no special type, *ER* estrogen receptor, *PgR* progesterone receptor, *HR* hormone receptor

*Parameters for which a *p*-value (from a χ^2^ test for independence) below 1% was observed, denoting a lack of independence between histopathological parameters and intrinsic subtypes

### Intrinsic subtypes and IHC groups

**Fig. 2:**
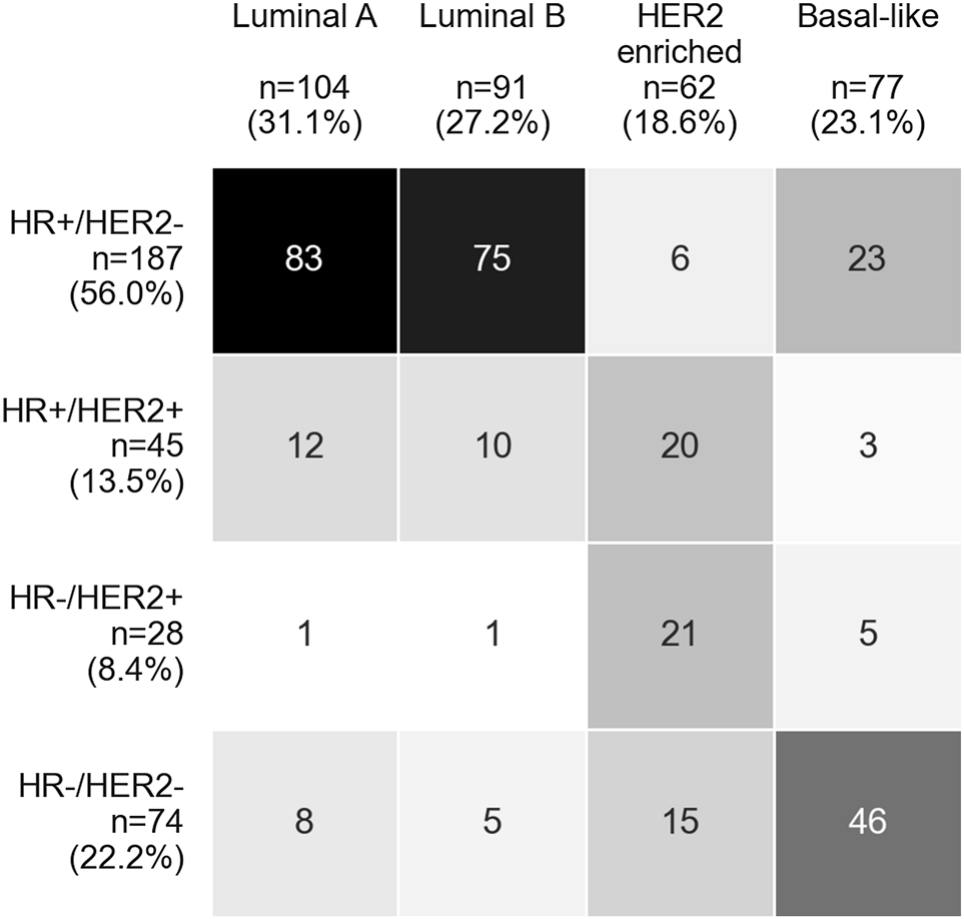
Color-coded crosstable of 334 BC tissue samples grouped according to IHC groups (rows) and intrinsic subtypes (columns). The cell color gradient indicates the relationship in terms of a strong discordance (white) to a strong concordance (black) between IHC and intrinsic subtype classifications. *HR* hormone receptor, *HER2* human epidermal growth factor receptor 2

A strong correlation was observed between intrinsic subtypes and IHC groups (*p*-value from χ^2^ test for independence < 0.001, Fig. 2). This association was driven mainly by the luminal subtypes, from which 81.6% (*n* = 158) were grouped as HR-positive and HER2-negative (Fig. 2, upper left). A multivariate regression allowed us to confirm the strong correlation (ORs > 20) between the luminal intrinsic subtypes and the HR+/HER2–IHC group, after adjusting for age, tumor size, histological type, tumor grade and Ki-67 proliferation index (Table 2).

**Table 2:** Results of multivariate logistic regression of clinical and histopathological parameters, taken as predictive variables for intrinsic subtypes

Parameters	Luminal A (*n* = 104)		Luminal B (*n* = 91)		HER2−enriched (*n* = 62)		Basal-like (*n* = 77)	
	OR (99% CI)	*p*-value	OR (99% CI)	*p*-value	OR (99% CI)	*p*-value	OR (99% CI)	*p*-value
Age group (years)								
< 50 (*n* = 201)	1.47 (0.68–3.16)	0.20	0.95 (0.46–1.97)	0.87	0.76 (0.30–1.92)	0.45	1.00 (0.43–2.31)	0.99
> 50 (*n* = 95)	Ref		Ref		Ref		Ref	
Tumor size								
T1 or T2 (*n* = 168)	Ref		Ref		Ref		Ref	
T3 or T4 (*n* = 126)	0.66 (0.30–1.44)	0.17	1.74 (0.84–3.62)	0.05	0.72 (0.28–1.88)	0.38	1.04 (0.45–2.42)	0.89
Histological type								
NST (*n* = 303)	1.36 (0.39–4.78)	0.53	1.88 (0.46–7.76)	0.25	0.50 (0.11–2.31)	0.24	0.65 (0.16–2.67)	0.43
Non-NST (*n* = 31)	Ref		Ref		Ref		Ref	
Tumor grade								
G1 or G2 (*n* = 140)	**3.19 (1.47–6.92)**	**1.21 × 10**^**–4**^	Ref		Ref		Ref	
G3 (*n* = 194)	Ref		1.16 (0.52–2.57)	0.63	1.45 (0.50–4.14)	0.37	**3.43 (1.26–9.34)**	**1.54 × 10**^**–3**^
Ki-67 proliferation index								
Low (< 20%) (*n* = 132)	**3.65 (1.72–7.72)**	**9 × 10**^**–6**^	Ref		Ref		Ref	
High (≥ 20%) (*n* = 202)	Ref		1.64 (0.76–3.56)	0.10	1.50 (0.54–4.13)	0.31	2.40 (0.95–6.09)	0.02
IHC group								
HR+/HER2−(*n* = 187)	**20.52 (1.36–309.24)**	**4.12 × 10**^**–3**^	**20.62 (1.43–297.79)**	**3.51 × 10**^**–3**^	Ref		2.88 (0.54–15.32)	0.10
HR+/HER2+(*n* = 45)	16.30 (0.94–281.70)	0.01	6.80 (0.41–111.73)	0.08	**22.62 (5.86–87.36)**	**2.74 × 10**^**–9**^	Ref	
HR−/HER2+(*n* = 28)	Ref		Ref		**88.30 (18.12–430.32)**	**3.16 × 10**^**–13**^	3.52 (0.45–27.62)	0.12
HR−/HER2−(*n* = 74)	3.89 (0.22–68.16)	0.22	1.86 (0.10–33.46)	0.58	**6.70 (1.77–25.39)**	**2.33 × 10**^**–4**^	**30.58 (5.49–170.50)**	**2.94 × 10**^**–7**^

*OR* odds ratio, *CI* confidence interval, *Ref* category taken as reference within each parameter for each test, *HER2* human epidermal growth factor receptor 2, *NST* no special type, *HR* hormone receptor

*Bold letters: cases with *p*-values < 0.01

Out of the 62 samples classified as HER2-enriched, 66.1% (*n* = 41) were defined as HER2–positive irrespective of HR status (Fig. 2). The results of a multivariate regression confirmed this, as HER2-enriched samples were significantly associated with a very high probability of being HER2-positive (independent of HR status, ORs > 20, Table 2). HER2-enriched samples were also associated with TNBC, although with a lower odds ratio (OR 6.7, 99% CI 1.77–25.39, Table 2).

The samples classified as basal-like had an increased probability of being TNBC (46 of 77 samples, 60%), as compared with triple-positive (HR+/HER2+) samples (Table 2).

### Intrinsic subtypes and histopathological parameters

**Fig. 3:**
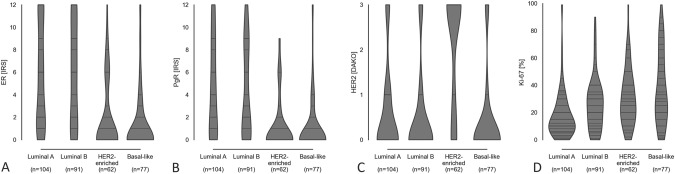
Scores of Estrogen Receptor, Progesterone Receptor, HER2 and Ki-67 Proliferation Index among intrinsic subtypes. The horizontal widths of the violin plots correspond to the frequency distribution of data points. Horizontal bars shown in the plots denote data available for those values. *ER* estrogen receptor, *PgR* progesterone receptor, *IRS* immune-reactive score, *ER* estrogen receptor, *PgR* progesterone receptor, *HER2* human epidermal growth factor receptor 2

Luminal A samples had a more than three-fold increased probability for favorable characteristics such as higher tumor differentiation (G1 or G2) and low Ki-67 proliferation index (OR 3.19, 99% CI 1.47–6.92 and OR 3.65, 99% CI 1.72–7.72, respectively, Table 2). Basal-like samples were associated with lower differentiation (G3; OR 3.43, 95% CI 1.26–9.34, Table 2). No further significant associations between intrinsic subtypes and histopathological parameters were found. A detailed analysis focused on the whole measured range of the prognostic parameters ER, PgR, HER2 and Ki-67 proliferation index is visualized in Fig. 3. While the IRS scores of ER and PgR spanned evenly between 0 and 12 for both luminal subtypes, these accumulated below IRS 2 for HER2-enriched and basal-like samples (Fig. 3A, B). This underlines the associations described between intrinsic subtypes and the expression of the steroid hormone receptors detected by IHC (see Table S2 for binary assessments). The violin plot of DAKO scores made the aforementioned association between HER2-enriched samples and positive HER2 status evident. The accumulation of scores > 2 was almost exclusively seen in HER2-enriched samples (Fig. 3C; Table S2). Similarly, specimens of low proliferation rates (< 20%) were more likely to be of the luminal A subtype (Fig. 3D; Table S2). However, low proliferation rates were also present in the other intrinsic subtypes. The highest proliferation rates were observed among basal-like samples.

## Discussion

In this prospective cohort of Ethiopian patients with BC, intrinsic PAM50-based subtypes of tumors were determined in addition to receptor status by immunohistochemistry (*n* = 334). Both methods had considerable similarities, especially for basal-like or triple-negative and HER2-enriched or HER2-positive types. The high proportion of patients below the age of 50 (approx. 60%) and large tumor sizes (T3 and T4, approx. 40%) differed from high-income settings. This probably resulted in the lower proportions of luminal A subtypes compared to Western cohorts.

### Pattern of subtypes

We reported in 2014 that two thirds of tumors from a patient cohorts from Addis Ababa had endocrine responsive disease [13]. Another cohort from rural Ethiopia showed more than half of the patients with positive hormone receptor status [6]. The third study published in 2018 again from Addis Ababa also reported 65% of receptor-positive disease [21]. Within the current study, immunohistochemistry as well as RNA-expression analysis were used to assess endocrine responsiveness. The results also revealed that even using RNA-expression based subtyping, more than half of the tumors were endocrine responsive. In detail, we found 31.1% luminal A, 27.2% luminal B, 18.6% HER2-enriched and 23.1% basal-like tumors. This is considerably shifted to the more aggressive subtypes compared, for example, to data from the United States (Nurses Health Study) that reported 46% luminal A, 18% luminal B, 14% HER2-enriched, 15% basal-like, and 8% normal-like subtypes [22]. Such direct comparison needs to be interpreted with care.

### Comparing with other ethnic groups

High quality data on tumor subtypes from the cancer genome atlas about tumors from African patients is lacking. Patients with African compared to European ancestry had a higher likelihood of basal-like (odds ratio OR 1.67) and HER2-enriched (OR 2.22) tumors [23]. African–American patients genetic background has been reported as predominantly from West Africa [24].

Geographic variations have been observed across Africa concerning the composition of different hormone receptor-based types no longer characterizing all tumors in Africa as having aggressive phenotypes. A large meta-analysis found that patterns of tumor subtypes in East Africa appear to be more favorable compared to other geographic regions such as West Africa [12].

### Other factors influencing subtype composition

Our convenient hospital cohort has specific features. The age composition as well as proportions of early and later stage disease may influence the overall proportions of subtypes. In general, cohorts from sub Saharan Africa seem to similarly represent a young population and commonly comprise of late stage tumors compared to Western settings. It was shown in a cohort from the United States that patients with luminal A tumors are on average 5–6 years older than patients with the other subtypes [25]. A comparison of receptor status between Sudanese and German patients also showed considerably more low-grade, receptor-positive tumors in older German women [26]. Therefore cohorts with fewer older patients lack these luminal tumors. Additionally, body composition has been reported to influence subtype. Obesity has been associated with higher risk of luminal A type breast cancer [27]. Since obesity is still rare in Ethiopia, less luminal A breast cancers are expected [28].

### Comparing subtypes and IHC

Assessing tumor biology with RNA-expression analysis is considered gold standard, at the same time immunohistochemistry has been used as basis to prove effectiveness of endocrine treatment. When comparing both methods, certain discrepancies can be seen. Consistent with previous reports [29], we have observed a substantial mismatch between the classification based on gene expression (PAM50 intrinsic subtypes) and on immunohistochemistry (IHC groups using ER, PgR and HER2). Still, hormone receptor-positive tumors were mainly classified as luminal subtypes. Therefore, immunohistochemistry results appear reliable as a basis for decision to advise endocrine treatment to the patient despite lack of standardized external quality assurance measures for tissue processing and immunohistochemistry performance [30].

In Ethiopia, as well as many other countries in Sub-Saharan Africa, basic immunohistochemistry is not always accessible to all patients. Even in capital cities, 15 out of 20 centers experienced frequent power cuts and four out of twenty had no immunohistochemistry in the country [31]. Due to the fact that also for Ethiopian patients’ routine immunohistochemistry is not always available, several confirmatory studies reported that half of patients have endocrine responsive disease (confirmed by RNA-expression as well as immunohistochemistry studies). This may provide clinicians more confidence in their decision process to offer endocrine treatment for patients with unknown receptor status.

### Individualized therapy

As a future perspective, multiplexed hybridization assay-based subtype determination via nCounter® can also support individualized therapy in Ethiopia. Since a large proportion of tumors are larger than 2 cm or lymph node-positive, without additional prognostic markers, according to the National Cancer Control Network (NCCN) harmonized guidelines, nearly all patients would need chemotherapy. As an example, utilizing PAM50-based subtypes, we were able to split the large group of patients with HR-positive and HER2-negative tumors into clinically relevant, more homogenous intrinsic subtypes. Patients with HR+/HER2 tumors have been regarded as an especially challenging BC group due to high variance concerning clinical outcome [32]. These patients include cases of low, intermediate and high risk of recurrence. Differentiating the HR+/HER2 group into luminal A and non-luminal A intrinsic subtypes increases the ability to assess recurrence risk in a more individualized manner, since chemotherapy is not recommended for patients with luminal A tumors [33, 34]. This information also enables clinicians to counsel patients on the importance of adherence to adjuvant endocrine therapy long-term and surveillance for local or distant recurrence.

### *Individualized *therapy: HER2*-enriched and basal-like types*

The subtype determination can also personalize treatment for patients with aggressive tumors. The PAM50 assay allowed the identification of 62 tumors as HER2-enriched, regardless of HER2 status. Using the IHC method, only 41 patients would get an anti-HER2 therapy. This means that the PAM50 assay leads to an approximately 50% incremented number of patients who would benefit from drugs suppressing the HER2 signaling pathway (anti-HER2 therapy). Our results are concordant with the seminal work of Perou and colleagues in the sense that two thirds of our samples classified as HER2-positive were found to be HER2-enriched (Table 1) [10].

Finally, 77 samples were classified as basal-like through the PAM50 assay. Out of these, only 46 (60%) were concordant with the triple-negative class via the IHC method. These samples are arguably the ones that would benefit most from chemotherapy, 40% of the basal-like samples are non-TNBC [10]. Conversely, approximately 38% of the TNBC tumors were non-basal-like. Thus, both PAM50 and IHC classification methods yield heterogeneous groups where personalized recommendations would improve by additional molecular subtyping as proposed before [35, 36].

### Strengths and limitations

The strength of the study includes multi-center involvement in Ethiopia and the application of multigene assays for gene expression profiling which is the gold standard for molecular classification as well as hormone receptor status by immunohistochemistry involving relatively large sample size. Both methods similarly showed a large share of endocrine responsive tumors. Certain limitations have to be taken into account. Firstly, the cohort is a convenient hospital-based sample and does not reflect the true pattern within the population. Given the resource-limited setting, population-based sample collection is not feasible. Secondly, Ethiopian patients vary considerably linguistically, ethnically and culturally within the country. Therefore results cannot be generalized within the country and a nation-wide study investigating distribution intrinsic subtypes and further exploration of tumor biology is essential to capture the large number of different ethnic groups in Ethiopia which could possibly help to have a broaden understanding of BC biology.

## Conclusion

To our knowledge, this is the first time that a large African BC patient group is characterized by molecular expression profiling. In summary, we confirmed previous immunohistochemistry results showing a considerable proportion of more than half the patients eligible for endocrine treatment. This allows utilization of a cost-effective treatment with very little side-effects that can be administered even at primary or secondary level health facilities. A study from Ethiopia proved that a strategy involving the specialized training of “cancer nurses” to support patients during their 5 year treatment can improve adherence [37].

Efforts are needed to provide sufficient access to immunohistochemistry service but in the meantime can encourage to utilization of endocrine treatment for patients with unknown receptor status. Within this project, colleagues from Ethiopia (ZD, MY, TA) received thorough training in tumor-banking, RNA-extraction and PAM50 subtyping. Additionally, during the time of the study and still ongoing is a larger training program to implement routine immunohistochemistry at Addis Ababa University involving weekly virtual meetings for case-discussions, several training courses in Ethiopia as well as in person mentorship in Germany. In 2022, consumables were provided and routine IHC for ER, PgR and HER2 determinations are performed in Ethiopia. Endpoint-PCR for research only was also implemented at Addis Ababa University as a step to further develop capacity in laboratory techniques. Since molecular methods are becoming available at lower prices, utilization of RNA-expression-based subtype assessment could become an option to optimize personalized treatment. One-step PCR technologies to assess basic receptor status are a possible compromise given the robustness of the methodology and at the same time relatively low cost availability.

## Supplementary Information

Below is the link to the electronic supplementary material.

Supplementary file1 (DOCX 18 kb)

Supplementary file2 (DOCX 18 kb)

## Data Availability

The data generated in this study are available within the article and its supplementary data files. Raw data were generated and processed from the authors and are available on request to the corresponding authors.
