# Supplementary material for: Intrinsic subtypes in Ethiopian breast cancer patient
Source: Breast Cancer Res Treat. 2022 Oct 25;196(3):495–504. doi: 10.1007/s10549-022-06769-z (PMC9633534; doi:10.1007/s10549-022-06769-z)
Supplement: Supplementary file 1 — Supplementary file1 (DOCX 18 kb) [file 10549_2022_6769_MOESM1_ESM.docx]

Supplementary Table S1: Distribution of clinical and histopathological parameters among IHC groups

| **Parameters** | **All** |  | **HR+/HER2− (%)** | **HR+/HER+ (%)** | **HR−/HER+ (%)** | **HR−/HER2− (%)** |
| --- | --- | --- | --- | --- | --- | --- |
|  | n=334 |  | n=187 (56.0) | n=45 (13.5) | n=28 (8.4) | n=74 (22.2) |
| **Age Group (years)** | |  |  |  |  |  |
| < 50 | 201 |  | 118 (58.7) | 30 (14.9) | 14 (7.0) | 39 (19.4) |
| ≥ 50 | 95 |  | 53 (55.8) | 10 (10.5) | 12 (12.6) | 20 (21.1) |
| unknown | 38 |  | 16 (42.1) | 5 (13.2) | 2 (5.3) | 15 (39.5) |
| **Tumor Size (pT)** | |  |  |  |  |  |
| T1 or T2 | 168 |  | 104 (61.9) | 21 (12.5) | 14 (8.3) | 29 (17.3) |
| T3 or T4 | 126 |  | 65 (51.6) | 19 (15.1) | 12 (9.5) | 30 (23.8) |
| unknown | 40 |  | 18 (45.0) | 5 (12.5) | 2 (5.0) | 15 (37.5) |
| **Histological Type** | |  |  |  |  |  |
| NST | 303 |  | 170 (56.1) | 43 (14.2) | 24 (7.9) | 66 (21.8) |
| Non-NST | 31 |  | 17 (54.8) | 2 (6.5) | 4 (12.9) | 8 (25.8) |
| **Tumor Grade** |  |  |  |  |  |  |
| G1 or G2 | 140 |  | 98 (70.0) | 13 (9.3) | 9 (6.4) | 20 (14.3) |
| G3 | 194 |  | 89 (45.9) | 32 (16.5) | 19 (9.8) | 54 (27.8) |
| **Estrogen Receptor Status** | |  |  |  |  |  |
| Positive (≥1) | 184 |  | 153 (83.2) | 31 (16.8) | 0 (0.0) | 0 (0.0) |
| Negative (<1) | 150 |  | 34 (22.7) | 14 (9.3) | 28 (18.7) | 74 (49.3) |
| **Progesterone Receptor Status** | |  |  |  |  |  |
| Positive (≥1) | 157 |  | 130 (82.8) | 26 (16.6) | 0 (0.0) | 1 (0.6) |
| Negative (<1) | 177 |  | 57 (32.2) | 19 (10.7) | 28 (15.8) | 73 (41.2) |
| **Hormone Receptor Status** | |  |  |  |  |  |
| Positive | 232 |  | 187 (80.6) | 45 (19.4) | 0 (0.0) | 0 (0.0) |
| Negative | 102 |  | 0 (0.0) | 0 (0.0) | 28 (27.5) | 74 (72.5) |
| **HER2 Status** |  |  |  |  |  |  |
| Negative | 261 |  | 187 (71.6) | 0 (0.0) | 0 (0.0) | 74 (28.4) |
| Positive | 73 |  | 0 (0.0) | 45 (61.6) | 28 (38.4) | 0 (0.0) |
| **Ki-67 Proliferation Index** | |  |  |  |  |  |
| Low (<20) | 132 |  | 86 (65.2) | 9 (6.8) | 11 (8.3) | 26 (19.7) |
| High (≥20) | 202 |  | 101 (50.0) | 36 (17.8) | 17 (8.4) | 48 (23.8) |

The percentages are given between brackets and they sum up to 100 in each row. HER2: Human Epidermal Growth Factor Receptor 2; NST: No special type; ER: Estrogen Receptor; PgR: Progesterone Receptor; HR: Hormone Receptor
