# Supplementary material for: Intrinsic subtypes in Ethiopian breast cancer patient
Source: Breast Cancer Res Treat. 2022 Oct 25;196(3):495–504. doi: 10.1007/s10549-022-06769-z (PMC9633534; doi:10.1007/s10549-022-06769-z)
Supplement: Supplementary file 2 — Supplementary file2 (DOCX 18 kb) [file 10549_2022_6769_MOESM2_ESM.docx]

Supplementary Table S2: Relationship between clinical or histopathological parameters and intrinsic subtypes, with proportions given within each row

| **Parameters** | **All** |  | **Luminal A** | **Luminal B** | **HER2-enriched** | **Basal-like** | **p-value** | |
| --- | --- | --- | --- | --- | --- | --- | --- | --- |
|  | **n=334**  **(100%)** |  | **n=104**  **(31.1%)** | **n=91**  **(27.2%)** | **n=62**  **(16.8%)**  **(18.6%)** | **n=77 (23.1%)** |  | |
| **Age Group (years)** |  |  |  |  |  |  | 0.67 | |
| < 50 | 201 (100%) |  | 66 (32.8%) | 58 (28.9%) | 33 (16.4%) | 44 (21.9%) |  | |
| ≥ 50 | 95 (100%) |  | 29 (30.5%) | 24 (25.3%) | 21 (22.1%) | 21 (22.1%) |  | |
| unknown | 38 (100%) |  | 9 (23.7%) | 9 (23.7%) | 8 (21.1%) | 12 (31.6%) |  | |
| **Tumor Size** |  |  |  |  |  |  | 0.01 | |
| T1 or T2 | 168 (100%) |  | 68 (40.5%) | 39 (23.2%) | 31 (18.5%) | 30 (17.9%) |  | |
| T3 or T4 | 126 (100%) |  | 29 (23%) | 41 (32.5%) | 23 (18.3%) | 33 (26.2%) |  | |
| unknown | 40 (100%) |  | 7 (17.5%) | 11 (27.5%) | 8 (20%) | 14 (35%) |  | |
| **Histological Type** |  |  |  |  |  |  | 0.45 | |
| NST | 303 (100%) |  | 94 (31%) | 86 (28.4%) | 54 (17.8%) | 69 (22.8%) |  | |
| Non-NST | 31 (100%) |  | 10 (32.3%) | 5 (16.1%) | 8 (25.8%) | 8 (25.8%) |  | |
| **Tumor Grade*** |  |  |  |  |  |  | **3.35 × 10^-11^** | |
| G1 or G2 | 140 (100%) |  | **71 (50.7%)** | 37 (26.4%) | 18 (12.9%) | 14 (10%) |  | |
| G3 | 194 (100%) |  | 33 (17%) | 54 (27.8%) | 44 (22.7%) | **63 (32.5%)** | |  |
| **Estrogen Receptor Status*** |  |  |  |  |  |  | **5.11 × 10^-16^** | |
| Positive (≥1%) | 184 (100%) |  | 77 (41.8%) | 69 (37.5%) | 18 (9.8%) | 20 (10.9%) |  | |
| Negative (<1%) | 150 (100%) |  | 27 (18%) | 22 (14.7%) | 44 (29.3%) | 57 (38%) |  | |
| **Progesterone Receptor Status*** |  |  |  |  |  |  | **6.99 × 10^-15^** | |
| Positive (≥1%) | 157 (100%) |  | 69 (43.9%) | 60 (38.2%) | 13 (8.3%) | 15 (9.6%) |  | |
| Negative (<1%) | 177 (100%) |  | 35 (19.8%) | 31 (17.5%) | 49 (27.7%) | 62 (35%) |  | |
| **Hormone Receptor Status*** |  |  |  |  |  |  | **4.43 × 10^-25^** | |
| Positive | 232 (100%) |  | 95 (40.9%) | 85 (36.6%) | 26 (11.2%) | 26 (11.2%) |  | |
| Pegative | 102 (100%) |  | 9 (8.8%) | 6 (5.9%) | 36 (35.3%) | 51 (50%) |  | |
| **HER2 Status*** |  |  |  |  |  |  | **7.56 × 10^-19^** | |
| Negative | 261 (100%) |  | 91 (34.9%) | 80 (30.7%) | 21 (8%) | 69 (26.4%) |  | |
| Positive | 73 (100%) |  | 13 (17.8%) | 11 (15.1%) | 41 (56.2%) | 8 (11%) |  | |
| **Ki-67 Proliferation Index*** |  |  |  |  |  |  | **2.48 × 10^-8^** | |
| Low (<20%) | 132 (100%) |  | **66 (50%)** | 31 (23.5%) | 17 (12.9%) | 18 (13.6%) |  | |
| High (≥20%) | 202 (100%) |  | 38 (18.8%) | 60 (29.7%) | 45 (22.3%) | 59 (29.2%) |  | |
| **IHC Group*** |  |  |  |  |  |  | **5.185 × 10^-40^** | |
| HR+/HER− | 187 (100%) |  | 83 (44.4%) | 75 (40.1%) | 6 (3.2%) | 23 (12.3%) |  | |
| HR+/HER+ | 45 (100%) |  | 12 (26.7%) | 10 (22.2%) | 20 (44.4%) | 3 (6.7%) |  | |
| HR−/HER+ | 28 (100%) |  | 1 (3.6%) | 1 (3.6%) | 21 (75%) | 5 (17.9%) |  | |
| HR−/HER2− | 74 (100%) |  | 8 (10.8%) | 5 (6.8%) | 15 (20.3%) | 46 (62.2%) |  | |

*Parameters for which a p-value (from a χ^2^ test for independence) below 1% was observed, denoting a lack of independence between histopathological parameters and intrinsic subtypes. HER2: Human Epidermal Growth Factor Receptor 2; NST: No special type; ER: Estrogen Receptor; PgR: Progesterone Receptor; HR: Hormone Receptor
